# Supplementary material for: Tribological Properties of Nitrate Graphite Foils
Source: Nanomaterials (Basel). 2024 Sep 15;14(18):1499. doi: 10.3390/nano14181499 (PMC11434555; doi:10.3390/nano14181499)
Supplement: Supplementary file 1 [file nanomaterials-14-01499-s001.zip › X-ray data/misorientation/01Hz/40-80/dens_16/TD/160.pdf]

Raw data

Sample Name:		X-ray	: Cu / 40 kV / 30 mA	Counter	: Scintillation counter
File	: 160.raw	Goniometer	: Ultima IV		
Comment	:	Attachment	: MPA-ML4(with gamma) for stress		
Date	: May-08-24 11:09:15	Filter	: K-beta filter	Scan mode	: Continuous
Operator	: Administrator	I.Monochro	: CBO	Scan speed	: 5.00 deg./min.
		C.Monochro	: Not installed	Sampling width	: 0.05 no unit
		DivSlit	: Open	Scan axis	: theta
		DivH.L.Slit	: 10.00mm	Scan range	: 10.00 -> 80.00 no unit
		SctSlit	: 2/3?‹|2/3deg.	Fixed angle	: 0.00 no unit
		RecSlit	: 0.30mm		
Memo	:				

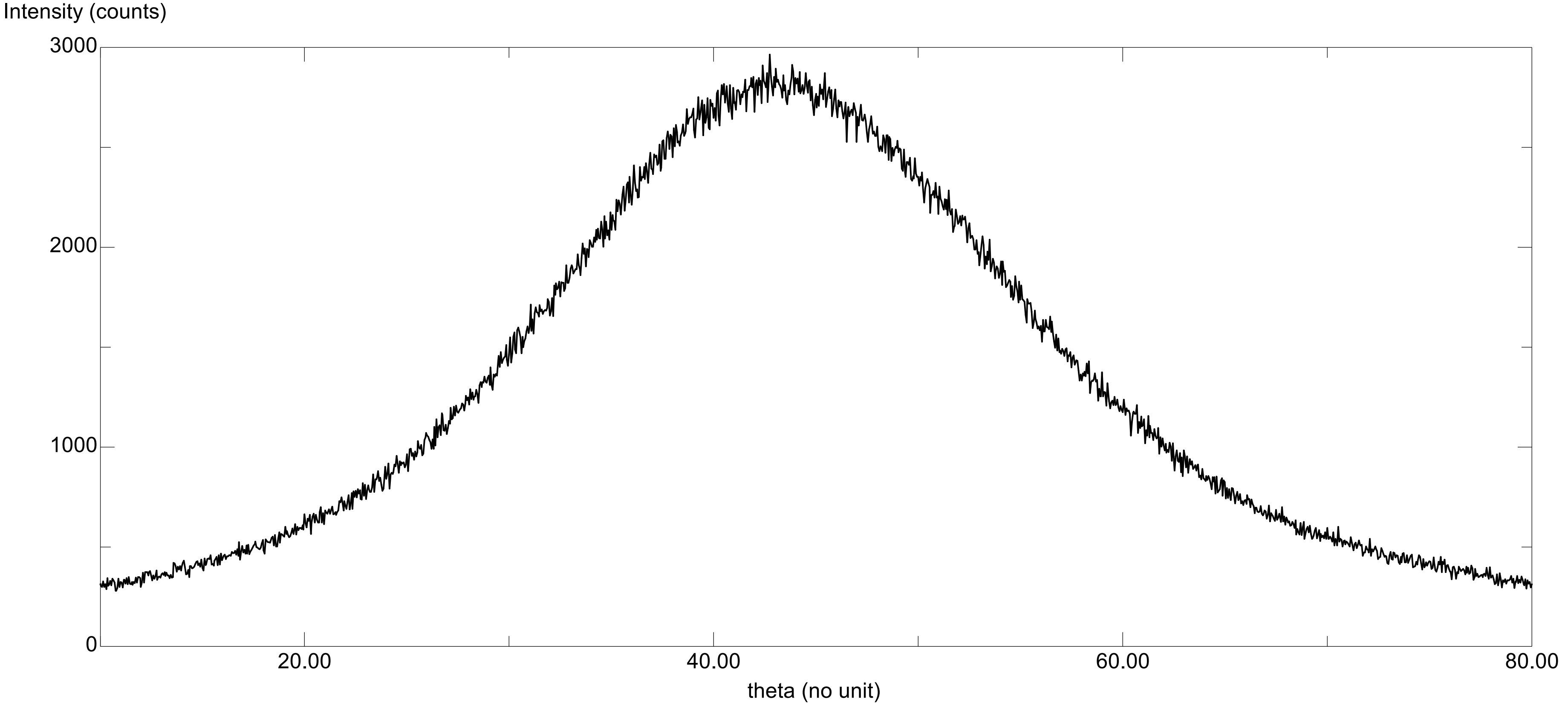

Integral Int. Calculation (History)

Sample Name: File : 160.raw Date : May-08-24 11:09:15 Operator : Administrator
Comment : Memo :
Peak calc. method: FW2/3M

h	k	l	Start	Stop	Peak	d-value	FWHM	Max. Int.		Gross int.	Integ.w	Integ. int.
0	0	0	10.00	no unit80.00	no unit43.46	no unit2.08	25.93	no unit2492	counts	1401573.00	28.117	70078.66

[Cut]
[Slit Correction]
[LP Corr.]

[Absorp. Corr.]
[Norm.]
[Smoothing] Method : Averaging Smoothing Points: 11
[BG subtraction] Method : Averaging at the end
Low angle : 5 High angle : 12
[Ka2 elimination]

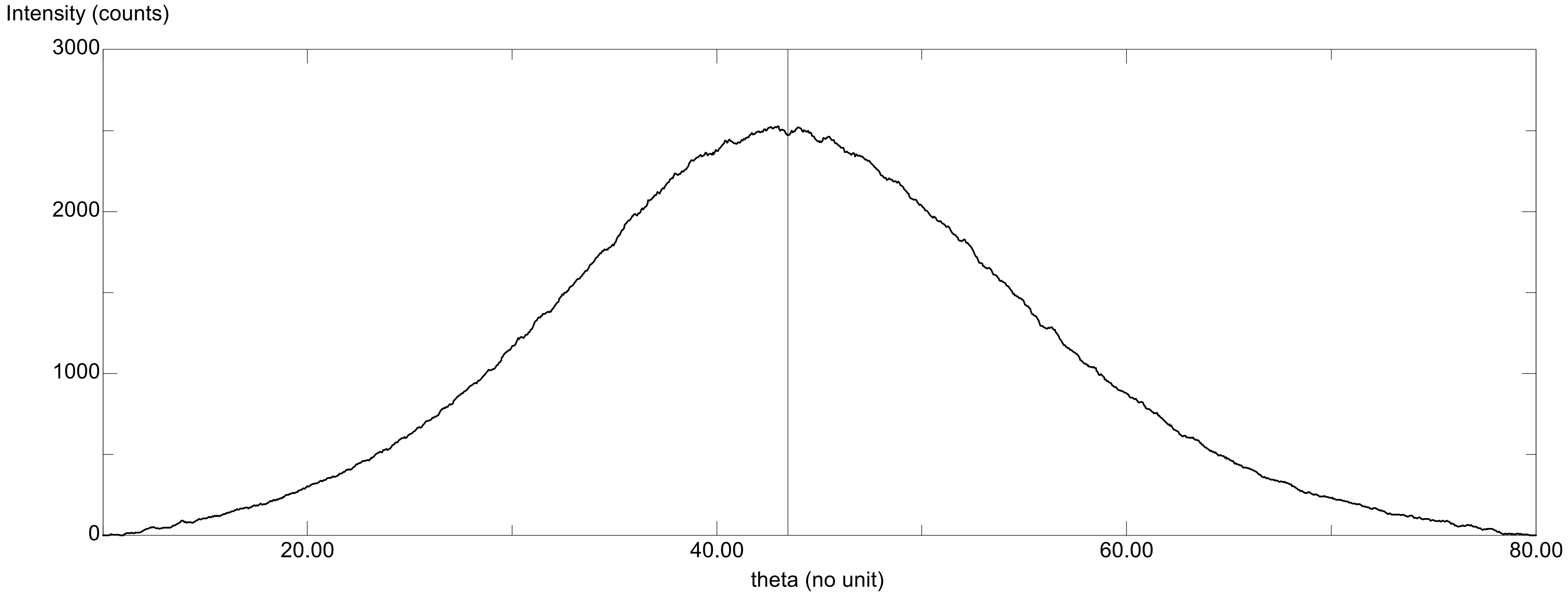

Integral int. sum table

Sample Name: File : 160.raw Date : May-08-24 11:09:15 Operator : Administrator
Memo :
Peak calc. method: FW2/3M

No.	h k l	Start	Stop	Peak	d-value	FWHM	Max. Int.	Gross int.	Integ.w	Integ. int.	Comment
1	0 0 0	10.00	80.00	43.46	2.08	25.93	2492	1401573.00	28.117	70078.66	
